# Effort but not Reward Sensitivity is Altered by Acute Sickness Induced by Experimental Endotoxemia in Humans

**DOI:** 10.1038/npp.2017.231

**Published:** 2017-11-15

**Authors:** Amelia Draper, Rebecca M Koch, Jos WM van der Meer, Matthew AJ Apps, Peter Pickkers, Masud Husain, Marieke E van der Schaaf

**Affiliations:** 1 Department of Experimental Psychology University of Oxford, Oxford, UK; 2 Department of Intensive Care Medicine, Radboud University Medical Center, Nijmegen, The Netherlands; 3 Department of Internal Medicine, Radboud University Medical Centre, Nijmegen, The Netherlands; 4 Donders Institute for Brain, Centre for Cognitive Neuroimaging, Cognition and Behaviour, Radboud University, Nijmegen, The Netherlands

## Abstract

Sickness behavior in humans is characterized by low mood and fatigue, which have been suggested to reflect changes in motivation involving reorganization of priorities. However, it is unclear which specific processes underlying motivation are altered. We tested whether bacterial endotoxin *E. coli* lipopolysaccharide (LPS) affected two dissociable constructs of motivational behavior, ie, effort and reward sensitivity. After familiarization with 5 effort levels, participants made a series of accept/reject decisions on whether the stake offered (1, 4, 8, 12, or 15 apples) was ‘worth the effort’ (10%, 27.5%, 45%, 62.5%, and 80% of maximal voluntary contraction in a hand-held dynamometer). Effort and reward levels were parametrically modulated to dissociate their influence on choice. Overall, 29 healthy young males were administered LPS (2 ng/kg; *n*=14) or placebo (0.9% saline; *n*=15). The effort-stake task, and self-reported depression and fatigue were assessed prior to LPS/placebo injection, 2 and 5 h post injection. Cytokines and sickness symptoms were assessed hourly till 8 h after LPS injection. LPS transiently increased interleukin-6 and tumor necrosis factor-*α*, sickness symptoms, body temperature and self-reported fatigue, and depression post injection relative to baseline and placebo. These changes were accompanied by LPS-induced decreases in acceptance rates of high-effort options, without significantly affecting reward sensitivity 2 h post injection, which were partially recovered 5 h post injection. We suggest that LPS-induced changes in motivation may be due to alterations to mesolimbic dopamine. Our behavioral paradigm could be used to further investigate effects of inflammation on motivational behavior in psychiatric and chronic illnesses.

## Introduction

Motivational symptoms such as apathy and fatigue are common in patients with psychiatric disorders including depression, schizophrenia, and bipolar disorder (Toomey *et al*, 1998). A growing field of research suggests that inflammation may contribute to these motivational symptoms (Felger and Treadway, 2017; Réus *et al*, 2015; Rosenblat *et al*, 2014). This is supported by observations of elevated levels of pro-inflammatory cytokines interleukin-6 (IL-6) and tumor necrosis factor-*α* (TNF*α*) in people who suffer from chronic major depression (Dowlati *et al*, 2010; Müller and Schwarz, 2007; Young *et al*, 2014) and observations of neuroinflammation in patients with bipolar disorder (Rao *et al*, 2009; Stertz *et al*, 2013; Yüksel and Öngür, 2010) and schizophrenia (Garver *et al*, 2003; Rosenblat *et al*, 2014).

Systemic inflammation in humans typically induces a cluster of non-specific symptoms (ie, sickness behavior) including fatigue, depression, and apathy (Dantzer, 2001). Studies of sickness behavior in animals suggest that these behavioral changes might be mediated by cytokine effects on the central nervous system (Dantzer *et al*, 2014; Felger and Miller, 2012; Swardfager *et al*, 2016). Following pathogen exposure, pro-inflammatory cytokines are released by activated immune cells to orchestrate the physiological immunologic response (Dembic, 2015). These pro-inflammatory cytokines also have a critical role in the regulation of immune influences on brain function (Kronfol and Remick, 2000) and have shown to affect dopamine function in mesolimbic brain regions (Neurauter *et al*, 2008; Capuron *et al*, 2012). Dopamine has repeatedly been associated with both reward and effort-based decision making, but it remains to be determined how inflammation affects effort and reward influences on behavior.

Several human studies have investigated reward learning and mesolimbic functioning after treatment with the inflammatory cytokine interferon alpha (INF*α*) or acute inflammation challenges. These studies demonstrated altered reward learning (Harrison *et al*, 2015) and reductions in reward-related ventral striatal activity, that was associated with inflammation-induced increases in depression, fatigue, and anhedonia (Capuron *et al*, 2012; Dowell *et al*, 2016; Eisenberger *et al*, 2010).

By contrast, research with animals suggest that inflammation affects effort expenditure, rather than reward processing (Larson *et al*, 2002; Larson, 2006; Nunes *et al*, 2014; Yohn *et al*, 2016). In a two-choice (high-effort/high-reward *vs* low-effort/low-reward) paradigm (Salamone *et al*, 1994), administration of IL-1*β* shifted rodent’s choice towards the low-effort/low-reward option. Importantly, reward sensitivity remained intact as high-reward preferences were unaffected (Nunes *et al*, 2014). Another study demonstrated that inflammation reduced the overall effort investment (ie, number of responses), whereas the better high-effort/high-reward option was still favored (Vichaya *et al*, 2014). A version of this latter paradigm was recently assessed in humans where participants chose between high-effort/high-reward and low-effort/low-reward options. Reward magnitude and probability was modulated (Lasselin *et al*, 2016). Although participants selected the high-effort/high-reward options at the same rate during inflammation compared to placebo, they selected a greater proportion of the high-effort options when the probability to win the reward was high. Thus, participants still performed the high-effort options during inflammation to gain a higher reward, suggesting that they are still reward sensitive.

Paradigms used to date have been limited in the dissociation of reward and effort influences as they typically compare high-reward/high-effort options with low-reward/low-effort options. Accordingly, in the current investigation, we aimed to test whether systemic inflammation differentially affects reward or effort processing in healthy human volunteers using a recently developed effort-stake choice paradigm (Bonnelle *et al*, 2015, 2016). In this paradigm, we parametrically modulate effort and reward choices by providing options with combinations of different levels of reward and effort, allowing us to dissociate effort and reward influences on choice.

Our second aim was to explore the relationship between changes in motivational behavior and changes in fatigue and depression or pro-inflammatory cytokine response. Informed by current literature highlighting the role of IL-1*β*, IL-6, and TNF*α* in various chronic conditions that express motivational symptoms, including in major depressive disorder (Dowlati *et al*, 2010; Young *et al*, 2014; (Engler *et al*, 2017; Felger and Treadway, 2017), rheumatoid arthritis (Roerink *et al*, 2017a); and cancer-related fatigue (Bower and Lamkin, 2013; Schubert *et al*, 2007; Smith *et al*, 2014; Wood and Weymann, 2013; Roerink *et al*, 2017a), as well as the effects of acute administration of IL-6 and IL-1*β* on animal behavior (Nunes *et al*, 2014; Vichaya *et al*, 2014; Bonsall *et al*, 2015; Yohn *et al*, 2016), we focused our investigation on these three pro-inflammatory cytokines.

## Materials and methods

### General Session Procedure

This study was part of a larger clinical trial at the department of intensive care medicine of the Radboudumc in Nijmegen in the Netherlands investigating the effects of human endotoxemia followed by the administration of a live-attenuated influenza vaccine ‘Fluenz’ on the immune response (ClinicalTrials.gov: NCT02642237). The human endotoxemia sessions took place 1 week before the administration of Fluenz and were therefore not confounded by this second part of the clinical trial. Participants received either *E. coli*-derived lipopolysaccharide (LPS) at a dose of 2 ng/kg or saline (0.9% NaCl) intravenously. They were randomly assigned to the LPS or placebo condition on the morning of testing by an unaffiliated research nurse and deblinding of conditions took place after all data had been collected. To control for individual differences in baseline performance, behavioral testing took place at three time points: session 1: 45 min before injection; session 2: 2 h post injection and; session 3: 5 h post injection. ‘Timing was based on previous experiences from our group showing that sickness symptoms are limited 2 h after LPS administration, whereas cytokine levels are still high (Kox *et al*, 2015; Leentjens *et al*, 2012).’ All study procedures were in accordance with the declaration of Helsinki, including the latest revisions and approved by the local medical ethics committee (CMO: 2015/2058).

### Participants

Thirty healthy, non-smoking Caucasian males aged 18–35 years old (median age 21; IQR: 20–23) without any medical/psychiatric history or current use of (prescription) drugs were recruited by the Radboud University Medical Centre Intensive Care Research Unit (see Table 1; Supplementary Materials for inclusion and exclusion criteria). All subjects were bachelor or master students from the local universities. To reduce potential variation related to gender or hormonal fluctuations in female menstrual cycle (Angele *et al*, 2014, Engler *et al*, 2016, Karshikoff *et al*, 2015; Moieni *et al*, 2015), only male subjects were used. Participants were asked to refrain from food (12 h) as well as caffeine and alcohol (24 h) before the LPS/placebo challenge. One volunteer was excluded due to vomiting that interfered with task performance during session 2 (LPS group: *N*=14, placebo group: *N*=15).

### Force-Level Familiarization

**Figure 1:**
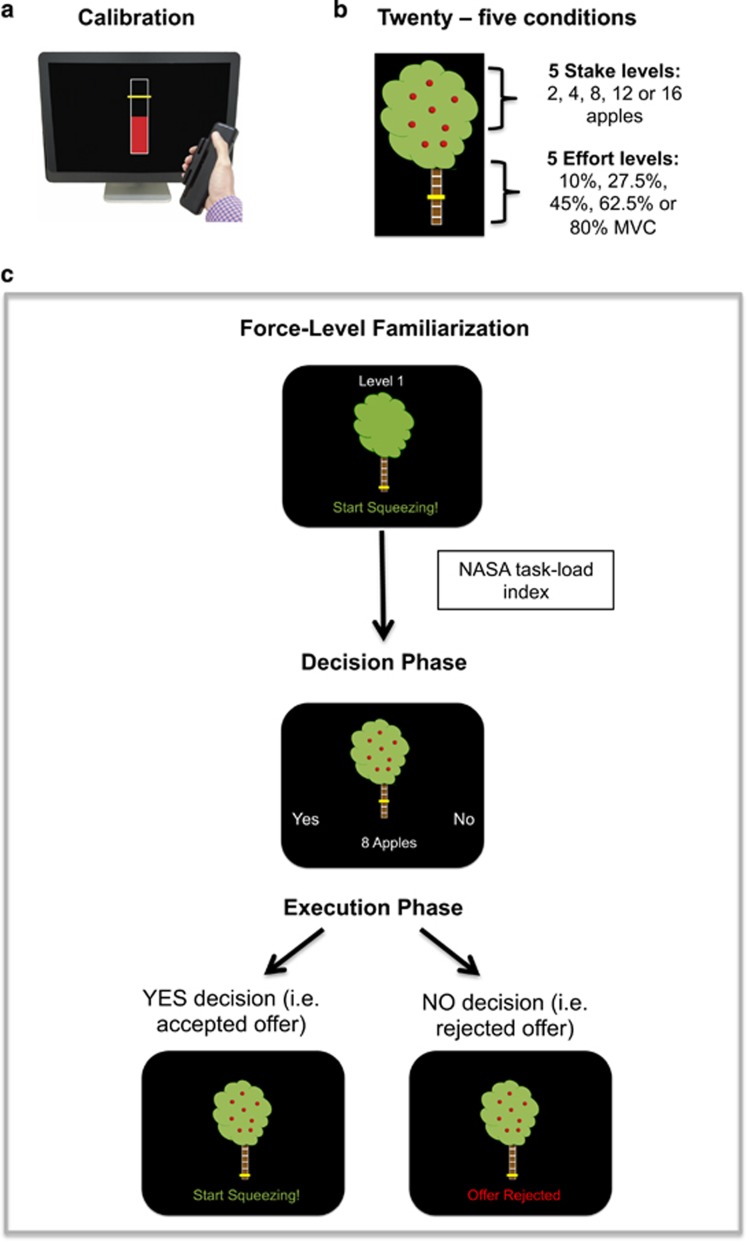
(a) Example of the feedback participants saw during calibration phase. (b) Representation of how effort and stake levels were presented to participants. Effort level was indicated by where the yellow line appeared on the tree’s trunk, starting at the bottom for effort level 1 and moving up to effort level 5 at the top. In the example pictured effort is set at level 3 corresponding to 45% MVC. Stake level is indicated by the number of apples on the tree, which ranged from 2 to 16 apples. In the example pictured stake is set at stake level 3 corresponding to 8 apples. (c) All the stages of the task that were repeated during each session. In force-level familiarization stage, participants had to reach each effort level twice, starting at effort level one (pictured) and moving up to effort level 5. They then completed the NASA task-load index questionnaire. During the decision phase, each of the 25 conditions were presented four times each in a pseudo-random order. Participants just had to select YES or NO to each offer. For the execution phase, 26 trials from the decision phase were randomly selected for the participant to perform. If an offer they had accepted (YES) was selected, they saw the command ‘start squeezing!’ and were able to attempt to reach the force level required to win the apples. If an offer they had rejected (NO) was selected the message ‘offer rejected’ appeared on the screen and they waited for the next trial to begin.

After estimation of maximum voluntary contraction (MVC) (see Supplementary Materials for details on apparatus and MVC estimation), the five effort levels were set as 10%, 27.5%, 45%, 62.5%, and 80% of each individual’s MVC, and represented as sections on the trunk of an apple tree (Figure 1b). Beginning at effort level 1, participants practiced squeezing to the required force and holding their grip at that force for 2 s (Figure 1c). The trunk of the tree filled up with red as the dynamometer was squeezed, and turned yellow as soon as the required force was reached. Each effort level was performed twice sequentially from level 1 to level 5 using the dominant hand. Force-level familiarizations were repeated at the start of each session to remind participants of the effort required for each level.

### Experimental Task

Participants were presented with a series of offers, in the form of the apple trees, and they had to select YES or NO, by lightly squeezing the right or left dynamometer, depending on if they evaluated the stake offered to be ‘worth the effort’. Twenty-five possible offers (all combinations of the 5 effort levels and 5 stake levels (1, 4, 8, 12, or 15 apples)) were each sampled four times in a pseudo-random order, totaling 100 trials in each session (Figure 1).

To make each judgment behaviorally relevant, participants were told that 26 decisions would be randomly selected for them to perform immediately following the decision phase. These 26 trials were then presented during an execution phase. If the offer was accepted, participants had 5 s to reach the required effort level and hold it for 2 s. If they were successful, they received feedback stating how many apples they had won. If the offer was rejected, the tree appeared on screen with the message ‘offer rejected’, meaning they were not able to attempt to win the apples offered (Figure 1c). Participants were told at the start of the day they would be rewarded based on the number of apples (worth 3 cents each) they gathered during this execution phase. Ten trials were practiced before session 1 to familiarize them with the stake/effort relationships. To control for changes in perceived task demand, participants performed a NASA task-load index questionnaire (Hart and Staveland, 1988) after each familiarization session. Participants rated temporal, physical, and mental demand; frustration, effort required and their performance for each effort level.

### Measurement of Sickness Behavior and Mood

Physical sickness symptoms were measured before LPS administration (T=0) and at 30-min intervals until 8 h after LPS administration (17 measures). Participants were asked to rate from 0 (absent) to 5 (very severe) the severity of six common symptoms: nausea, headache, muscle aches, back pain, shivers, and vomiting. Self-reported mood was assessed using the depression and fatigue subscales of the profile of moods state questionnaire (POMS (McNair *et al*, 1971)) at the start of each session (see Supplementary Materials for details on the subscale items).

### Measurement of Cytokines in Plasma

EDTA-anticoagulated blood was collected at: 1, 1.5, 2, 3, 4, 6, and 8 h after LPS administration, centrifuged (2000 *g*, 4 °C, 10 min), and stored at −80 °C until analysis. Plasma concentrations of cytokines of interest (TNF*α* and IL-6) were measured using a simultaneous (entered together in one batch) Luminex assay (R&D systems; Abingdon Science Park, UK, Human high sensitivity cytokine kit, catalog numbers LHSCM000, LHSCM210, LHSCM206, www.rndsystems.com). Statistical analyses to calculate plasma concentrations were performed using Graphpad Prism version 5.0 (Graphpad Software, San Diego, CA, USA). Lower detection limits in plasma and intra-assay coefficients of variation (C.V.) were; 0.22 pg/ml (C.V. 1.54%) for TNF*α* and <0.86 pg/ml (C.V. 0.92%) for IL-6. We initially also aimed at assessing IL1-Ra and IL1-b because of the suggested association between IL1 and fatigue (Roerink *et al*, 2017a). Unfortunately, luminex assays of IL1-Ra failed and measures were considered unreliable as concentrations of IL1-Ra exceeded the upper detection limit (>15 296 pg/ml, C.V.=0.046%). IL-1*β* was excluded from analyses because plasma concentrations did not exceed lower detection limits at 2 h post injection (0.79 pg/ml, C.V.=0.30%).

## Statistical analysis

### Behavioral Task

The percentage of accepted offers for each of the 25 conditions (5 effort and 5 stake levels) was the key variable for each participant. We first tested whether LPS induced a change in choice behavior between session 1 and session 2. Acceptance rates were entered into a 5 (effort level) × 5 (stake level) × 2 (time) × 2 (groups) AVOVA. On the basis of our hypothesis, we were specifically interested in whether LPS affected effort and/or stake sensitivity. Accordingly, our tests of interests were the effort × time × stake × group interaction and, if significant, the effort × time × group and stake × time × group interactions. If LPS induced a significant change in choice behavior, we subsequently ran an ANOVA comparing session 1 and session 3 to test whether the LPS-induced changes in choice behavior recovered to baseline.

These analyses were repeated with generalized estimating equation (GEE) using a binary logistic model and exchangeable working correlation structure, which is better suited for binomially distributed categorical outcomes. Effort level, stake level, and trial (1–100) were mean centered and entered as continuous variables, session and group (LPS/placebo) were entered as factors, and the model contained all main effects and interactions.

### Subjective Measures, Cytokines, and Physiology

A total score for sickness symptoms was calculated for each subject at each time point. Febrile response and sickness symptoms were entered into a repeated-measures ANOVA with the factors time (17 levels) and group. Plasma concentrations of cytokines (TNF*α* and IL-6) were entered into a repeated-measures ANOVA with the factors time (8 levels) and group.

Total scores for depression and fatigue were calculated as mean score on the POMS subscales for each session. A repeated-measures ANOVA with the factors time (3 levels) and group was performed for fatigue and depression scores separately. Similar to our behavioral analysis, we first assessed LPS-induced changes between session 1 and 2. If significant, we assessed whether changes recovered to baseline by comparing session 1 and 3. *Post-hoc* Bonferroni-corrected independent *t*-tests were calculated, where appropriate.

### Relationship Between Effort/Stake Sensitivity and Mood and Cytokines

To assess whether LPS-induced changes in behavior were associated with changes in mood and/or cytokines, we first calculated the individual levels of effort and reward sensitivity via a binomial logistic regression in Matlab with effort and stake level as predictors and the decisions (yes/no) as dependent variable for each subject. The *β*’s were standardized by dividing by the standard error to minimize the impact of inflated *β*’s (Apps *et al*, 2015) (Supplementary Table S2). Change (session 2−session 1) in the standardized *β*’s were used to assess relationships with LPS-induced changes in mood and/or cytokines. Two stepwise multiple regressions were used with change in effort sensitivity and change in stake sensitivity as dependent variable. Change in depression, change in fatigue, peak concentration of IL-6, and peak concentration of TNF*α* were entered as predictor variables in both regressions. All regressions were computed using only data from the LPS group. Statistical threshold for all stepwise multiple regression analyses were Bonferroni-corrected for multiple comparisons.

### Control Analyses

To assess whether LPS effects were explained by alterations in perceived physical demand, we assessed LPS effects on total scores of the NASA task-load index questionnaire (sum of each section) using a repeated-measures ANOVA with the factors effort, time, and group. We additionally assessed LPS effects on the NASA task-load index subscales ‘physical demand’ (How physically demanding was the task?) and ‘effort’ (how hard did you have to work to accomplish the task?) using repeated-measures ANOVA with the factors effort, time and group, and on the sickness symptom ‘muscle aches’ using repeated-measures ANOVA with the factors time and group. When no groups’ differences were found, we tested whether LPS effects on behavior remained significant when the NASA subscales or the sickness symptom ‘muscle aches’ were added as covariate into the ANOVA on acceptance rates.

Finally, we assessed the relationship between standardized *β*’s from the binomial logistic regression and other variables that may confound the behavioral results (ie, total sickness symptoms, muscle aches, febrile response, NASA physical demand, and NASA effort) within the LPS group using Pearson’s correlation analysis. Total sickness symptoms and febrile response could not be added as covariate in the ANOVA because they were significantly affected by LPS (see ‘Results’) and therefore violate the assumption of homogeneity of regression slopes (Miller and Chapman, 2001).

## Results

### Behavioral Task

**Figure 2:**
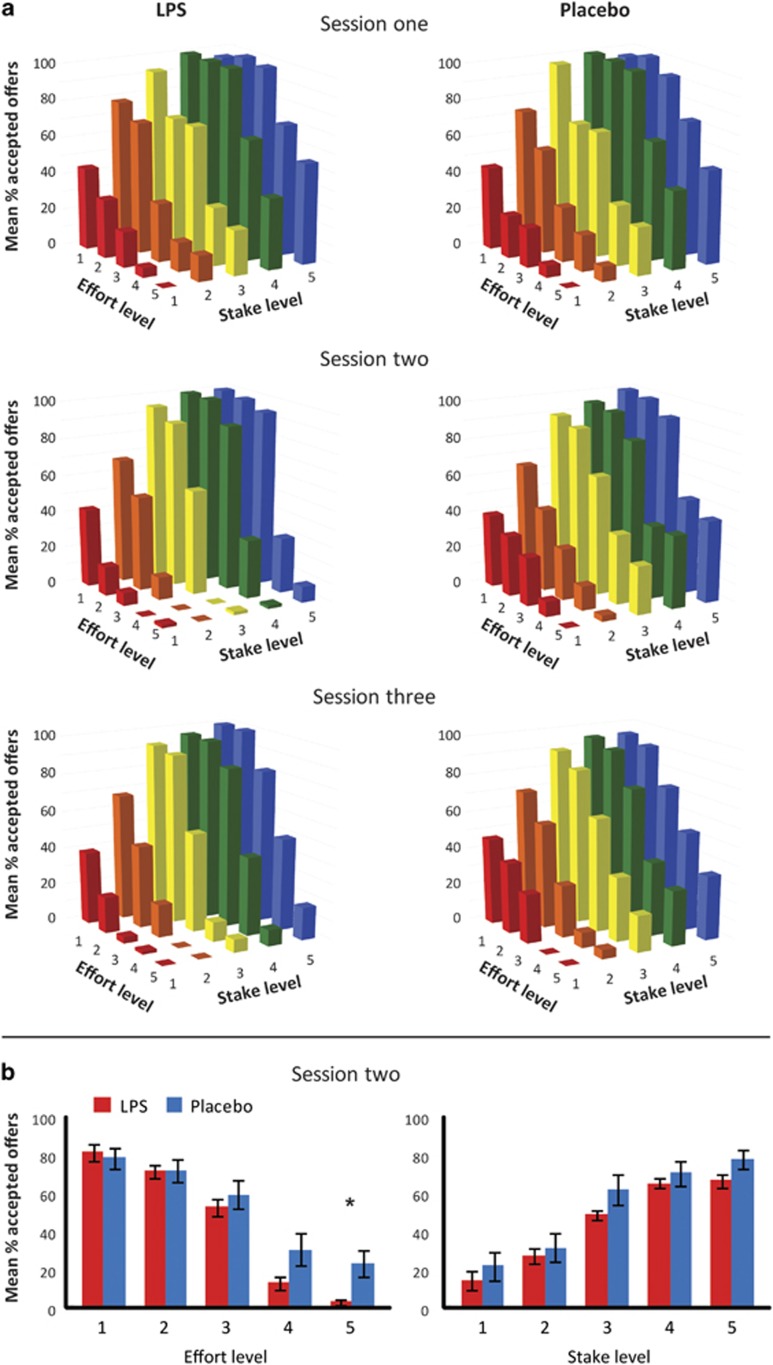
(a) The mean percentage of accepted offers during the decision phase for each of the 25 conditions (5 effort × 5 stake). Left column is results from the LPS group, right column is results from the placebo group. Top row is results from session 1, middle row from session 2, and bottom row is from session 3. (b) The mean percentage of accepted offers during session 2 collapsed across each effort (left) and stake (right) level. Error bars represent the standard error of the mean. *p<0.05 in a Student’s *t*-test.

Results are presented in Figure 2 and Supplementary Tables S1 and S2. As shown previously in healthy people (Bonnelle *et al*, 2015, 2016), there were significant main effects of effort and stake on acceptance rates, demonstrating that both groups were sensitive to the effort and stake manipulations (acceptance rates increase with higher stake and lower effort levels) (stake: F_(4,108)_=131.2, *p*<0.001, effort: F_(4,108)_=117.2, *p*<0.001).

LPS effects on stake differed significantly from LPS effects on effort (stake × effort × time × group: (F_(16,432)_=1.81, *p*=0.028), with significant group effects on effort-related acceptance rates (effort × time × group: F_(4,108)_=3.2, *p*=0.016), but not stake-related acceptance rates (stake × time × group: F_(4,108)_=0.4, *p*>0.7). Breakdown of the interaction by group revealed that effort-related acceptance rates were reduced in the LPS group (stake × effort × time: F_(16,208)_=3.12, *p*<0.001; effort × time: F_(4,56)_=4.93, *p*=0.002; stake × time: F_(4,56)_=1.77, *p*=0.15), but not in the placebo group (stake × effort × time: F_(16,208)_=1.17, *p*=0.29; effort × time: F_(4,56)_=1.13, *p*=0.34; stake × time: F_(4,56)_=2.28, *p*=0.11). Further breakdown of the effort × time × group interaction by session revealed that there was no between group difference at session 1 on any of the effort levels (all *p*>0.05), and that the LPS group accepted less offers than the placebo group for the highest effort level during session 2 (80%: *T*_(27)_=−2.695, *p*=0.012; 62.5%: *T*_(27)_=−1.843, *p*=0.076). This between group difference on effort was not present during session 3, compatible with partial recovery (effort × stake × time × group: F_(16,432)_=1.295, *p*=0.196; effort × time × group (F_(4,108)_=0.366, *p*=0.833; stake × time × group (F_(4,108)_=0.999, *p*=0.411).

These findings were confirmed with GEE (stake × effort × time × group: *β*=−0.026, SD=0.13, *p*=0.043; effort × time × group: *β*=−0.85, SD=0.22, *p*<0.001; stake × time × group: *β*=0.36, SD=0.19, *p*=0.062). Breakdown of the interaction by group revealed that effort but not stake-related acceptance rates were reduced in the LPS group (stake × effort × time: *β*=0.24, SD=0.11, *p*=0.021; effort × time: *β*=0.75, SD=0.20, *p*<0.001; stake × time: *β*=−0.12, SD=0.13, *p*=0.35), whereas the trend observed for the stake × time × group interaction was driven by a trend in the placebo group (stake × effort × time: *β*=−0.02, SD=0.08, *p*=0.81; effort × time: *β*=−0.14, SD=0.10, *p*=0.17; stake × time: *β*=0.27, SD=0.14, *p*=0.053). Further breakdown of the effort × time × group interaction by session revealed that there was a between group difference at session 2 (stake × effort × group: *β*=−0.31, SD=0.13, *p*<0.016; effort × group: *β*=1.03, SD=0.25, *p*<0.001; stake × group: *β*=−0.46, SD=0.24, *p*=0.053) but not at session 1 (stake × effort × group: *β*=−0.066, SD=0.23, *p*=0.42) or session 3 (stake × effort × group: *β*=−0.184, SD=0.12, *p*=0.14) (Supplementary Tables S3 and S4).

Total rewards obtained in the execution phase ranged between €2.67–€6.42 for session 1, €1.25–€6.39 for session 2, and €1.41–€6.39 for session 3 and did not differ between groups (all *p*>0.05) (Supplementary Table S2). All subjects were able to successfully perform all effort levels twice before each session, indicating that LPS did not affect the ability to perform high-effort trials.

### Subjective Measures, Cytokines, and Physiology

**Figure 3:**
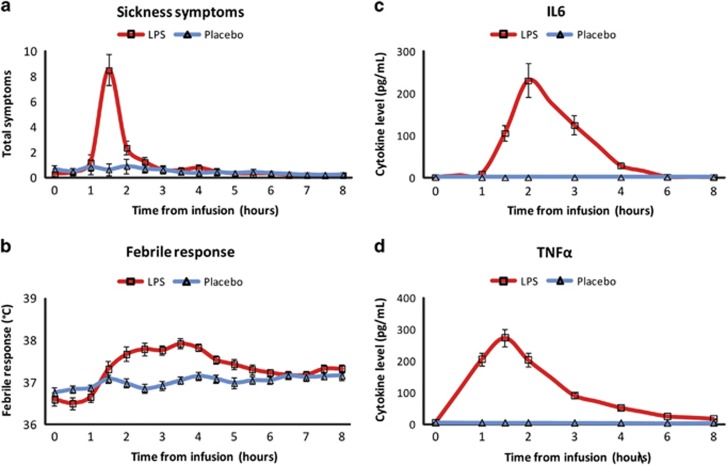
(a) Time course of mean total sickness symptoms scores. (b) Time course of febrile response. (c) Time course of mean plasma cytokine level for IL-6. (d) Time course of mean plasma cytokine level for TNF*α*. Error bars represent the standard error of the mean.

LPS, but not placebo, induced an increase in sickness symptoms (group × time: F_(1,26)_=18.9, *p*<0.001), which peaked at 1.5 h post injection. Importantly, sickness symptoms were significantly higher in the LPS group relative to the placebo group at session 2, whereas no group differences were observed at session 1 prior to injection, nor at session 3 at 5 h post injection (session 1 *T*_(27)_=1.37, *p*>0.1; session 2: *T*_(27)_=2.15, *p*<0.05; session 3: *T*_(27)_=0.30, *p*>0.7, Figure 3a; Supplementary Table S5).

LPS resulted in a 1±0.6 **°**C (mean±SD) increase in temperature (F_(16,432)_=13.4, *p*<0.001) and marked increases in all cytokines of interest at session 2 (IL-6: F_(7,189)_=32.48, *p*<0.001; TNF*α*: F_(7,189)_=88.83, *p*<0.001) (Figure 3b–d). Temperature and cytokines reduced back to baseline by 8 h from injection. The placebo group showed no change in any of the cytokines throughout the whole recorded period.

**Figure 4:**
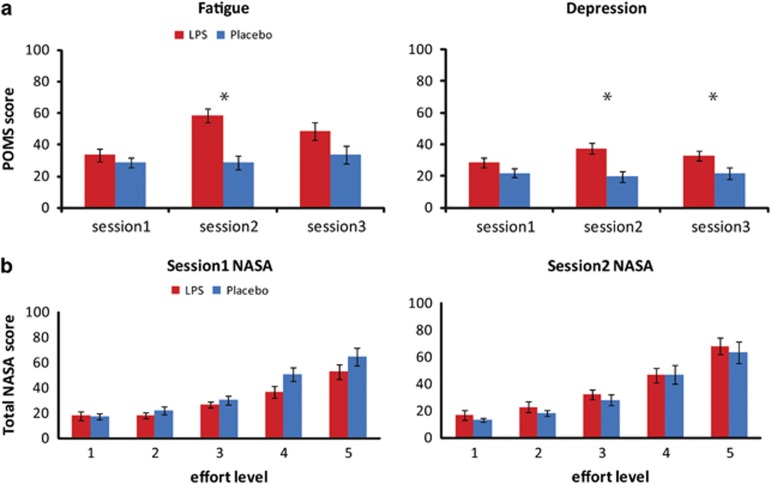
(a) Mean profile of mood state (POMS) score for the fatigue (left) and depression (right) subscales for each session. (b) Total NASA task-load index score for each effort level for session 1 (left) and session 2 (right). Error bars represent standard error of the mean. *p<0.05 in a Student’s *t*-test.

LPS affected self-reported depression and fatigue levels (depression time × group: F_(1,27)_=10.997, *p*=0.003; fatigue time × group: F_(1,27)_=23.6, *p*<0.001). Specifically, the LPS group reported feeling significantly more depressed and fatigued than the placebo group during session 2 (depression: *T*_(27)_=3.609, *p*<0.001; fatigue: *T*_(27)_=4.806, *p*<0.001), but not session 1 (depression: *T*_(27)_=1.517, *p*=0.141; fatigue: *T*_(27)_=0.915, *p*=0.368). The time × group interaction effect was no longer significant for fatigue or depression when looking at session 3 *vs* session 1 (depression: *T*_(27)_=1.579, *p*=220; fatigue: *T*_(27)_=2.477, *p*<0.127). However, direct comparisons revealed that the LPS group remained significantly more depressed than the placebo group during session 3, but only marginally more fatigued (depression: *T*_(27)_=2.221, *p*=0.035; fatigue: *T*_(27)_=1.942, *p*=0.063) suggesting only partial recovery for fatigue (Figure 4a).

### Relationship Between Effort/Stake Sensitivity and Mood and Cytokines

No relationship between changes in motivational behavior and mood or cytokines were observed: POMS scores for depression and fatigue, IL-6 or TNF did not remain in the stepwise multiple regression analyses as significant predictors for LPS-induced change in effort or stake sensitivity (all *p*>0.1, Supplementary Table S6).

### Control Analyses

The LPS group did not differ from the placebo group on the total NASA score or any of the NASA subscales (no significant interaction effects, minimum *p*>0.05, Figure 4b; Supplementary Table S5) indicating that LPS did not affect perceived (physical) demand of the requested effort levels. LPS effects on muscle aches did not differ between the groups (F_(1,27)_=1.11, *p*=0.30).

Adding the factor ‘physical demand’ as covariates to the main analyses did not affect our main result (F_(16,400)_=1.802, *p*=0.029), and no relationships were observed between ‘physical demand’ and effort sensitivity (*r*_(14)_=0.34, *p*=0.02). However, adding the subscale ‘effort’ as covariate to the main analyses resulted in a trend for the effort × stake × time interaction (F_(16,400)_=1.65, *p*=0.054). Change in NASA effort was also marginally correlated with change in effort sensitivity (*r*=0.369, *p*=0.053) (Supplementary Table S6).

Adding muscle aches a covariate in the analysis of decisions did not affect our main result (F_(16,400)_=1.70, *p*=0.044, Supplementary Table S1). No relationships between LPS effects on behavior and muscle aches, total sickness symptoms, and febrile response were observed (all *p*>0.05, Supplementary Table S6).

## Discussion

In this study, we used a relatively new paradigm that parametrically modulates offers with respect to stake and the effort required to obtain that reward (Bonnelle *et al*, 2015, 2016). We demonstrate that experimentally induced endotoxemia using LPS in humans reduces otherwise healthy participant’s willingness to accept high-effort options, without significantly altering reward sensitivity.

It has been suggested that sickness behavior is an adaptive motivational state that involves reprioritization of the costs and benefits of expending effort, rather than simply being general physical weakness (Dantzer, 2001). Our results support this claim: LPS reduced acceptance rates of high-effort options, whereas the ability to perform the task was not changed. All participants were able to successfully perform the effort levels during a familiarization phase prior to their decisions and participants reported no differences in perceived demand to perform the task as indexed by the NASA task-load index. This suggests that the changes we observed are due to altered motivation, rather than a change in physical strength or ability.

Changes in motivation are common across a broad range of psychiatric and medical conditions (Fervaha *et al*, 2015; Kostić and Filippi, 2011; Sinha *et al*, 2013; Starkstein and Pahissa, 2014). There is increasing evidence that inflammation may have an important role in development of amotivated states (Leboyer *et al*, 2012; Miller *et al*, 2009; Potvin *et al*, 2008; Réus *et al*, 2015; Bonaccorso *et al*, 2001; Capuron *et al*, 2012; Majer *et al*, 2008; Wichers *et al*, 2005). Here, we found that LPS increased cytokines and concomitantly altered motivational behavior and mood supporting the concept that inflammation could have a role in development of symptoms such as depression and fatigue (Dowell *et al*, 2016; Engler *et al*, 2017; Felger and Treadway, 2017; Karshikoff *et al*, 2017; Miller and Raison, 2016).

However, we could not demonstrate direct relationships between LPS-induced behavioral changes and cytokine concentrations or mood. This could be due to our small sample size, lacking the power to detect such associations. In addition, the immune manipulation we used (LPS) induced robust increases with little variation in multiple cytokines that strongly interact with one another, making it difficult to disentangle which cytokine is responsible for the observed change in behavior. Future studies that induce more variation, eg, by using different dosages or selectively stimulate or inhibit cytokines could provide more insight on the role of specific cytokines on change in behavior. Although many studies have suggested relationships between motivational behavior and mood, actual reports of these relationships have been limited (Lasselin *et al*, 2016; Salamone *et al*, 2016). One reason for this could be that the POMS was not sensitive enough to detect subtle alterations in depressive mood and fatigue. Alternatively, because our task dissociated between the sub-constructs of motivation (effort and reward) (Berridge *et al*, 2009), it may have captured behavior that is not necessarily reflected by the subjective reports of fatigue and depression (Karshikoff *et al*, 2017).

To the best of our knowledge, there has only been one previous human study that investigated effort and reward processing during inflammation (Lasselin *et al*, 2016). That pioneering investigation used two-option choices (high-reward/high-effort *vs* low-reward/low-effort) and showed that after LPS the high-effort option was still favored, more so when the probability of gaining the reward was also high. There was no difference between LPS and placebo conditions in the number of high-effort choices. Although our results may at first appear to contradict these findings, both studies demonstrated that reward sensitivity was not affected by LPS. In addition, the two studies differed in important ways.

First, in Lasselin *et al*’s forced-choice design, participants had to perform an action on each trial (meaning they updated their experience of the effort options), and they chose to perform the high-effort actions to gain a higher reward at the same rate as after placebo. In our design, participants only had the option to perform actions they considered ‘worth the effort’ for the reward offered, ie, they could choose to do nothing, which led to a reduction in choice of high-effort options during inflammation. Furthermore, our paradigm parametrically modulated effort and reward, rather than offering two-option choices. In this way, our task allowed participants to gain the high rewards at a lower effort level, meaning gaining the largest reward was not contingent on putting in the highest effort. Second, the previous human study (Lasselin *et al*, 2016) had a probability dimension, the likelihood of gaining the reward on high-effort trial was variable, an aspect which we did not test. Our task was simpler: reward was always given if the participant reached the effort level required. Finally, the previous investigation tested their participants 4 h post LPS, while we tested participants 2 h post LPS, which might be why our subjects were more effort sensitive. Indeed, the alterations in effort sensitivity were partly recovered 5 h after LPS.

One potential mechanisms through which cytokines can affect motivational behavior is through interference with brain dopamine function (Felger and Treadway, 2017). Indeed, effort- and reward-related components of motivated behavior have consistently been linked to dopamine signaling (Chong *et al*, 2015; Skvortsova *et al*, 2017; Wardle *et al*, 2011). For example, using a variation of the task reported here, Chong *et al* 2015 observed that patients with Parkinson’s disease, who often experience motivational symptoms, were willing to put in more effort to gain rewards when they were ON dopaminergic drugs compared to when they were OFF them. Animal work has also shown that dopamine depletion in the nucleus accumbens causes similar changes in effort-based choice behavior as observed here (Randall *et al*, 2012). However, previous neuroimaging work has mainly focused on LPS effects on reward-related processes, showing reduced reward-related signals in the ventral striatum (Capuron *et al*, 2012; Eisenberger *et al*, 2010; Harrison *et al*, 2015). Given the partial dissociable brain networks of effort and reward processing (Klein-Flügge *et al*, 2016; Skvortsova *et al*, 2014), the neural alterations related to LPS effects on effort-based choice remained to be determined. We unfortunately do not have brain imaging data to compare to previous work (Capuron *et al*, 2012; Eisenberger *et al*, 2010; Harrison *et al*, 2015). Future studies are needed to better dissociate the neural mechanisms of inflammation effects on effort- and reward-related processes.

Insights into immune-to-brain pathways gained from acute immune manipulation studies are important for better mechanistic understanding of motivational deficits in psychiatric conditions, and could lead to new pharmaceutical targets. Indeed, dopamine enhancing drugs like methylphenidate or levodopa can reverse inflammation effects (Yohn, 2016; Bonsall, 2015; Felger *et al*, 2015) and has some effects on reducing fatigue in humans (Blockmans *et al*, 2006; Kerr *et al*, 2012; Mendonça *et al*, 2007; Minton *et al*, 2011). It remains to be investigated whether other dopamine manipulations would also reverse inflammation effects on motivational symptoms. Here we show that our task is sensitive to immune manipulation on dissociable sub-constructs of motivational behavior (reward and effort) in humans. This paradigm is therefore promising for further human research on immune-mediated changes in motivational behavior, and for testing of pharmacological targets to treat motivational symptoms. These could include targets at the immunological level by inhibiting pro-inflammatory cytokines; a method that has reduced fatigue symptoms in some medical conditions (Omdal and Gunnarsson, 2005; Roerink *et al*, 2017b), but also targets at the central level, affecting dopamine function, for patients groups in which immune-alterations are less prominent (Roerink *et al*, 2017a).

This study has several limitations. First, the sample size was small, lacking power to detect relevant associations between individual immune responses, motivated behavior, and mood. Second, we used a relatively high dose of LPS (2 ng/kg). This inherently also induce sickness symptoms that could affect the blindness of the conditions and potentially confound task performance. Although we show that our effects are specific to effort and not reward-based decisions, and that sickness symptoms were not directly correlated with effort-based choice within the LPS group, it is unfortunately statistically impossible to dissociate sickness symptoms from behavioral alterations, as these factors are not independent (Miller and Chapman, 2001). Simply lowing the dose might not help as this inherently also reduces the behavioral effects, while still inducing significant increases in sickness symptoms (Benson *et al*, 2017). Instead, future studies might potentially be able to dissociate these factors, eg, by assessing effects of co-administration with centrally acting drugs that do not affect sickness symptoms. Finally, we tested only men, whereas clinical work indicates that motivational symptoms such as depression are more prone in woman (Altemus *et al*, 2014). In addition, several studies highlight the importance of sex differences in immune–brain interactions that likely mediate the LPS effects on mood and behavior (Engler *et al*, 2016; Karshikoff *et al*, 2015; Moieni *et al*, 2015). This, therefore, limits generalizability of our results.

In summary, experimental endotoxemia reduced, otherwise, healthy participant’s willingness to engage in high-effort options, while reward sensitivity was not significantly altered. This change in motivation was not due to the task being perceived as more effortful. Endotoxemia concomitantly induced an increase in subjective reports of depression and fatigue. The behavioral paradigm used in this study provides a human model to further investigate brain mechanisms underlying inflammation effects on motivational behavior. A better understanding of these mechanisms in humans will be important for further development and testing of pharmaceutical targets to treat motivational deficits in psychiatric disorders.

## Funding and disclosure

This research was funded by a Wellcome Trust Principal Fellowship awarded to Prof Masud Husain and supported by the Oxford NIHR BRC and CRF. This work was also supported by an EFRO Grant (2011-013287). The authors declare no conflict of interest.

## Figures and Tables

**Table 1:** Characteristics of Participants

	**LPS group (*n*=14)**		**Placebo group (*n*=15)**		
	**Median**	**IQR**	**Median**	**IQR**	***p*-value (between groups** ***t*-test)**
Age (years)	21	20–23	22	19–23	0.90
Height (cm)	180	178–188	186	178–189	0.16
Weight (kg)	75	70–84	79	71–87	0.65
BMI (kg/m)^2^	23	20–26	23	22–25	0.93

Abbreviation: IQR, interquartile range.

All participants were male.
