# Supplementary material for: Effort but not Reward Sensitivity is Altered by Acute Sickness Induced by Experimental Endotoxemia in Humans
Source: Neuropsychopharmacology. 2017 Nov 15;43(5):1107–18. doi: 10.1038/npp.2017.231 (PMC5854801; doi:10.1038/npp.2017.231)
Supplement: Supplementary Information [file npp2017231x1.docx]

**Supplementary materials:**

*Apparatus*

Stimulus was programmed using the Psychtoolbox (<http://psychtoolbox.org>) in MATLAB (the Math Works Inc., USA) and presented on a laptop computer, while participants sat up straight in their hospital bed. Participants responded by squeezing one of two TSD121B-MRI hand dynamometers (BIOPAC Systems Inc., USA), which have a sample rate of 500 Hz. The force signal from the hand dynamometers was digitized and fed into the stimulus laptop in real time, and recorded in MATLAB. The experimental task used in this study was a modified version of the apple gathering task described previously (Bonnelle *et al*, 2015, 2016).

*Estimation of maximum voluntary contraction*

At the beginning of *Session 1* participants were asked to squeeze the dynamometer as strongly as they could using their dominant hand. Visual feedback was provided of a black bar filling up with red from the bottom, the level of which corresponded to the amount of force being applied to the dynamometer (Figure 1A). The procedure was repeated three times, with a yellow line, positioned on the bar at the level of maximum force recorded thus far, serving as a target to exceed. Maximum voluntary contraction (MVC) was estimated as the maximum force recorded from all three attempts. It was kept constant throughout all sessions for each individual.

*Profile of Moods*

The subscales depression (8 items) and fatigue (6 items) were presented as visual analogue scales with opposing moods at each extreme, presented on a tablet computer using qualtrics online survey software (<https://www.qualtrics.com>). The items were comparable to the 4-item scales used in other LPS studies (e.g. Eisenberger et al., 2010).

*Complete exclusion criteria:*

- Female
- Pre-existent lung disease, including asthma
- A history of allergic rhinitis
- Use of any medication
- Current smoker or more than 5 pack-year history
- Use of recreational drugs within 21 days prior to start of the study
- Use of caffeine or alcohol within 1 day prior to start of the study
- Surgery or trauma with significant blood loss or blood donation within 3 months prior to start of the study
- Participation in another clinical trial within 3 months prior to start of the study
- Frequent nosebleeds
- Recent nasal or otologic surgery
- (suspected) influenza infection during the last year
- Clinically significant acute (febrile) illness or a common cold within four weeks prior to start of the study
- History of frequent vaso-vagal collapse or of orthostatic hypotension History, signs or symptoms of cardiovascular disease.
- History of allergic reaction to Fluenz™, eggs / gelatin / gentamicin
- History of Guillain-Barré Syndrome
- Cardiac conduction abnormalities on the ECG consisting of a 2^nd^ degree atrioventricular block or a

complex bundle branch block.

- Hypertension (defined as RR systolic > 160 or RR diastolic > 90).
- Hypotension (defined as RR systolic < 100 or RR diastolic < 50).
- Renal impairment (defined as plasma creatinin >120 μmol/l).
- Liver function abnormality: alkaline phosphatase>230 U/L and/or ALT>90 U/L
- CRP > 20 mg/L, WBC > 12x10^9^/L

|  | stake*time*group | | effort*time*group | | effort*stake*time*group | |
| --- | --- | --- | --- | --- | --- | --- |
| covariate | F | p | F | p | F | p |
| No covariate | 0.476 | 0.753 | 3.184 | 0.016* | 1.81 | 0.028* |
| Muscle aches | 0.439 | 0.741 | 2.671 | 0.036* | 1.697 | 0.044* |
| NASA physical demand | 0.446 | 0.775 | 2.289 | 0.065^ | 1.802 | 0.029* |
| NASA effort | 0.492 | 0.741 | 2.284 | 0.065^ | 1.65 | 0.054^ |

**TableS1:** ANOVA and ANCOVA results on behavioral choice data without and with potential confounding factors included as covariate. * p<.05, ^p<.1

|  | LPS |  |  | Placebo |  |  |
| --- | --- | --- | --- | --- | --- | --- |
| Session | 1 | 2 | 3 | 1 | 2 | 3 |
| *% yes Effort* |  |  |  |  |  |  |
| 10% of MVC | 82.5(4.6) | 81.1(3.2) | 79.3(4.1) | 82.3(4.4) | 78(5.8) | 79.3(6.7) |
| 27,5 % of MVC | 80.4(3.4) | 71.1(4.5) | 70.4(5.9) | 74(4) | 71.3(7.5) | 72.3(9.3) |
| 45% of MVC | 64.3(5.2) | 52.1(3.9) | 48.6(5.7) | 62.3(5.8) | 58.7(8.6) | 53(7.7) |
| 62,5% of MVC | 38.2(7.8) | 12.5(1.6) | 21.1(3.8) | 39.7(7.4) | 30.3(7.1) | 27.3(7) |
| 80% of MVC | 26.8(7.9) | 2.9(5) | 6.8(4.1) | 26(7.1) | 23(7.3) | 18(6.6) |
| *% yes reward* |  |  |  |  |  |  |
| 1 apples | 20.4(6.5) | 13.6(4) | 12.9(5) | 19.3(5.9) | 21.7(7.3) | 22.3(7) |
| 4 appels | 42.5(7.5) | 26.4(2.5) | 26.1(4.4) | 37.7(6.8) | 31(8) | 33.7(7.7) |
| 8 apples | 63.9(5.2) | 48.6(2.6) | 51.1(4.5) | 63(5.5) | 61.3(6.2) | 58(7) |
| 12 apples | 81.1(4.5) | 64.6(3.5) | 66.1(4.6) | 81.3(4.5) | 70(5.1) | 66.3(6.9) |
| 15 apples | 84.3(4.9) | 66.4(1.6) | 70(3.8) | 83(4.6) | 77.3(7.1) | 69.7(7) |
| *T-value of binomal logistic regression* | |  |  |  |  |  |
| Effort | -2.9(0.5) | -3.9(0.3) | -3.9(0.2) | -2.6(0.5) | -2.6(0.5) | -2.7(0.5) |
| Stake | 3.4(0.4) | 3.3(0.3) | 3.5(0.3) | 3.4(0.4) | 3(0.4) | 2.3(0.5) |
| *Reward obtained in execution phase* | |  |  |  |  |  |
| Total number of apples | 150(11) | 115(4) | 124(9) | 147(10) | 134(15) | 133(15) |

**Table S2:** mean (SE) of % accepted offers for each effort and reward level per group and session, mean T-values (SE) of effort and reward sensitivity calculated from the binomial logistic regressions per group and session and the total number of apples worth 3 cents each (SE) obtained in the execution phase.

|  |  | Session2 vs Session1 | | |  | Session3 vs Session1 | | |  |  |  |  |
| --- | --- | --- | --- | --- | --- | --- | --- | --- | --- | --- | --- | --- |
|  |  | beta | SD | p |  | beta | SD | p |  |  |  |  |
| ***LPS vs PLACEBO*** |  |  |  |  |  |  |  |  |  |  |  |  |
| *Repeated variables* | Session | 0.141 | 0.5781 | 0.002 |  | 1.031 | 0.3899 | 0.008 |  |  |  |  |
|  | Trial | 1.029 | 0.3326 | <.001 |  | -0.008 | 0.0035 | 0.019 |  |  |  |  |
|  | Effort | -1.924 | 0.182 | <.001 |  | -0.476 | 0.1903 | <.001 |  |  |  |  |
|  | Stake | 1.362 | 0.1857 | <.001 |  | 1.243 | 0.2042 | <.001 |  |  |  |  |
| *Effects of interest* | Stake*effort*time*group | **-0.263** | **0.1297** | **0.043** |  | **-0.194** | **0.1532** | **0.205** |  |  |  |  |
|  | Effort*time*group | **-0.851** | **0.2213** | **<.001** |  | -0.394 | 0.2963 | 0.184 |  |  |  |  |
|  | Stake*time*group | **0.357** | **0.191** | **0.062** |  | 0.422 | 0.2722 | 0.121 |  |  |  |  |
| ***LPS group*** |  |  |  |  |  |  |  |  |  |  |  |  |
| *Repeated variables* | Session | .1.042 | 0.3218 | 0.001 |  | 1.04 | 0.3758 | 0.006 |  |  |  |  |
|  | Trial | -0.016 | 0.0033 | <.001 |  | -0.008 | 0.0034 | 0.016 |  |  |  |  |
|  | Effort | -1.948 | 0.1822 | <.001 |  | -0.130 | 0.1856 | <.001 |  |  |  |  |
|  | Stake | 1.378 | 0.1907 | <.001 |  | 1.26 | 0.1937 | <.001 |  |  |  |  |
| *Effects of interest* | Stake*effort*time | **0.248** | **0.1072** | **0.021** |  | **0.055** | **0.1363** | **0.687** |  |  |  |  |
|  | Effort*time | **0.752** | **0.2033** | **<.001** |  | 0.269 | 0.2667 | 0.268 |  |  |  |  |
|  | Stake*time | **-0.123** | **0.1321** | **0.353** |  | 0.001 | 0.1896 | 0.997 |  |  |  |  |
| ***PLACEBO group*** |  |  |  |  |  |  |  |  |  |  |  |  |
| *Repeated variables* | Session | 0.437 | 0.2756 | 0.113 |  | 0.713 | 0.3868 | 0.065 |  |  |  |  |
|  | Trial | 0.001 | 0.0015 | 0.887 |  | -0.001 | 0.0019 | 0.509 |  |  |  |  |
|  | Effort | -0.948 | 0.1658 | <.001 |  | -0.962 | 0.1684 | <.001 |  |  |  |  |
|  | Stake | 0.948 | 0.1282 | <.001 |  | 0.769 | 0.1531 | <.001 |  |  |  |  |
| *Effects of interest* | Stake*effort*time | **-0.018** | **0.0755** | **0.813** |  | -0.143 | 0.0755 | 0.057 |  |  |  |  |
|  | Effort*time | -0.141 | 0.1029 | 0.17 |  | -0.154 | 0.1769 | 0.383 |  |  |  |  |
|  | Stake*time | 0.268 | 0.1388 | 0.053 |  | 0.473 | 0.2114 | 0.025 |  |  |  |  |
| **Table S3.** Results from generalized estimating Equation (GEE) analysis using a binary logistic model with choice (yes/no) as dependent variable and effort (5 levels), stake (5 levels), session (2 levels), trial (100 trials) as predictor variables, comparing the LPS and Placebo groups (upper part) and per group separately (lower parts). Effects of interest and relevant post-hoc tests are presented in bold. | | | | | | | | |  |  |  |  |
|  |  | Session1 | | |  | Session2 | | |  | Session3 | | |
|  |  | beta | SD | p |  | beta | SD | p |  | beta | SD | p |
| ***LPS vs PLACEBO*** |  |  |  |  |  |  |  |  |  |  |  |  |
| *Repeated variables* | group | -0.553 | 0.6522 | 0.397 |  | 0.244 | 0.4818 | 0.612 |  | 0.379 | 0.5653 | 0.502 |
|  | Trial | -0.013 | 0.0055 | 0.016 |  | -0.016 | 0.0034 | <.001 |  | -0.008 | 0.0034 | 0.016 |
|  | Effort | -1.222 | 0.1509 | <.001 |  | -1.966 | 0.1827 | <.001 |  | -1.509 | 0.1861 | <.001 |
|  | Stake | 1.281 | 0.173 | <.001 |  | 1.39 | 0.1979 | <.001 |  | 1.27 | 0.1894 | <.001 |
| *Effects of interest* | Stake*effort*group | **0.066** | **0.0828** | **0.427** |  | **0.305** | **0.1268** | **0.016** |  | **0.184** | **0.1243** | **0.139** |
|  | Effort*group | 0.136 | 0.2248 | 0.544 |  | **1.031** | **0.252** | **<.001** |  | 0.546 | 0.2547 | 0.032 |
|  | Stake*group | -0.069 | 0.2317 | 0.766 |  | **-0.456** | **0.2361** | **0.053** |  | -0.5 | 0.2444 | 0.041 |

**Table S4.** Results from generalized estimating Equation (GEE) analysis per session separately using a binary logistic model with choice (yes/no) as dependent variable and effort (5 levels), stake (5 levels), trial (100 trials) and group (LPS/placebo) as predictor variables. Effects of interest and relevant post-hoc tests are presented in bold.

| Condition | LPS |  | |  | Placebo | | |  |  | F-value | |  |
| --- | --- | --- | --- | --- | --- | --- | --- | --- | --- | --- | --- | --- |
| Session | 1 | 2 | | 3 | 1 | | | 2 | 3 | T2vsT1 | | T3vsT1 |
| *POMS:* |  |  | |  |  | | |  |  |  | |  |
| Depression | 28.4(3) | 37.5(3.4) | | 32.8(3.1) | 22(3) | | | 19.7(3.5) | 22(3.7) | 10.997* | | 1.597 ns |
| Fatigue | 33.5(3.9) | 58.5(4.4) | | 48.7(5.4) | 28.9(3.1) | | | 28.7(4.3) | 33.6(5.5) | 23.6* | | 2.48 ns |
| *NASA task load index* | | | | | | | | | | | | |
| Mental | 4.1(0.6) | | 5.2(1) | 5.2(0.9) | | 5.7(0.8) | 4.5(0.8) | | 4.5(0.8) | | 3.385 ns |  |
| Physical | 6.5(0.6) | | 7.3(0.6) | 7.4(0.5) | | 7.9(0.7) | 7.6(0.7) | | 8(0.8) | | 1.584 ns |  |
| Temporal | 4.9(0.6) | | 5.9(0.7) | 6(0.8) | | 5.4(0.8) | 5.1(0.9) | | 5.1(0.8) | | 0.966 ns |  |
| Performance | 4.3(0.5) | | 5.7(0.8) | 5.9(0.8) | | 5.3(0.6) | 5(0.8) | | 5.5(0.9) | | 1.121 ns |  |
| Effort | 6.4(0.7) | | 7.4(0.8) | 7.6(0.7) | | 8.4(0.9) | 7.1(0.8) | | 7.9(0.8) | | 3.181 ns |  |
| Frustration | 8(1.1) | | 6(0.9) | 5.1(0.8) | | 8.6(1.2) | 4.9(0.7) | | 5.3(0.8) | | 1.056 ns |  |
| Total | 30.6(3.1) | | 37.5(4.1) | 37.2(4.1) | | 37.3(3.6) | 34.1(3.9) | | 36.3(4.1) | | 3.238 ns |  |
| *Sickness* |  | |  |  | |  |  | |  | |  |  |
| Total | 0.3(0.2) | | 2.4(0.5) | 0.3(0.2) | | 0.7(0.2) | 0.9(0.6) | | 0.4(0.2) | | 6.408* | - 1. ns |

**Table S5**. Mean (SE) of subjective reports on mood, NASA task load index and sickness symptoms, F-values represent group*time interactions, POMS = Profile of Moods *=p<.05, ns = not significant.

|  | effort |  | stake |  |
| --- | --- | --- | --- | --- |
|  | r/beta | p | r/beta | p |
| IL6 | 0.34 | 0.236 | -0.337 | 0.238 |
| TNF | 0.307 | 0.286 | -0.356 | 0.221 |
| POMS depression | -0.11 | 0.708 | 0.256 | 0.419 |
| POMS Fatigue | -0.306 | 0.288 | 0.235 | 0.419 |
| Muscle aches | 0.351 | 0.219 | -0.256 | 0.376 |
| Sickness Total | -0.042 | 0.886 | 0.332 | 0.246 |
| Febrile response | 0.367 | 0.196 | -0.092 | 0.754 |
| NASA physical demand | 0.278 | 0.153 | -0.124 | 0.53 |
| NASA effort | 0.369 | 0.053 | -0.253 | 0.195 |

**TableS6**. Standardized beta’s or Pearson’s correlations between LPS induced changes on effort/stake sensitivity and cytokines, mood, sickness symptoms and perceived task demand (NASA) within the LPS group.
